# The novel uncompetitive NMDA receptor antagonist esmethadone (REL-1017) has no meaningful abuse potential in recreational drug users

**DOI:** 10.1038/s41398-023-02473-8

**Published:** 2023-06-07

**Authors:** Megan J. Shram, Jack E. Henningfield, Glen Apseloff, Charles W. Gorodetzky, Sara De Martin, Frank L. Vocci, Frank L. Sapienza, Thomas R. Kosten, Jeff Huston, August Buchhalter, Judy Ashworth, Ryan Lanier, Franco Folli, Andrea Mattarei, Clotilde Guidetti, Stefano Comai, Cedric O’Gorman, Sergio Traversa, Charles E. Inturrisi, Paolo L. Manfredi, Marco Pappagallo

**Affiliations:** 1 Altreos Research Partners, Toronto, Ontario Canada; 2 Pinney Associates, Bethesda, MD USA; 3 Ohio Clinical Trials, Columbus, OH USA; 4 Relmada Therapeutics, Coral Gables, FL USA; 5 Consultant in Pharmaceutical Medicine, Kansas City, MO USA; 6 Department of Pharmaceutical and Pharmacological Sciences, University of Padova, Padova, Italy; 7 Friends Research Institute, Baltimore, MD USA; 8 The Drug and Chemical Advisory Group LLC, Fairfax, VA USA; 9 Baylor College of Medicine, MD Anderson Cancer Center, University of Houston, Houston, TX USA; 10 Department of Health Science, University of Milan, Milan, Italy; 11 Child and Adolescent Neuropsychiatry Unit, Department of Neuroscience, Bambino Pediatric Hospital, IRCCS, Rome, Italy

**Keywords:** Diseases, Depression

## Abstract

Esmethadone (REL-1017) is the opioid-inactive dextro-isomer of methadone and a low-affinity, low-potency uncompetitive NMDA receptor antagonist. In a Phase 2, randomized, double-blind, placebo-controlled trial, esmethadone showed rapid, robust, and sustained antidepressant effects. Two studies were conducted to evaluate the abuse potential of esmethadone. Each study utilized a randomized, double-blind, active-, and placebo-controlled crossover design to assess esmethadone compared with oxycodone (Oxycodone Study) or ketamine (Ketamine Study) in healthy recreational drug users. Esmethadone 25 mg (proposed therapeutic daily dose), 75 mg (loading dose), and 150 mg (Maximum Tolerated Dose) were evaluated in each study. Positive controls were oral oxycodone 40 mg and intravenous ketamine 0.5 mg/kg infused over 40 min. The Ketamine study included oral dextromethorphan 300 mg as an exploratory comparator. The primary endpoint was maximum effect (E_max_) for Drug Liking, assessed using a bipolar 100-point visual analog scale (VAS). A total of 47 and 51 participants completed the Oxycodone Study and the Ketamine Study, respectively (Completer Population). In both studies, esmethadone doses ranging from therapeutic (25 mg) to 6 times therapeutic (150 mg) had a meaningful and statistically significantly (*p* < 0.001) lower Drug Liking VAS E_max_ compared with the positive control. Results were consistent for all secondary endpoints in both studies. In both studies, all doses of esmethadone were statistically equivalent to placebo on Drug Liking VAS E_max_ (*p* < 0.05). In the Ketamine Study, Drug Liking VAS E_max_ scores for esmethadone at all tested doses were significantly lower vs. dextromethorphan (*p* < 0.05) (exploratory endpoint). These studies indicate no meaningful abuse potential for esmethadone at all tested doses.

## Introduction

Major depressive disorder (MDD) is the second leading cause of disability and chronic disease burden in the United States, among all medical conditions [1]. According to data from the National Epidemiologic Survey on Alcohol and Related Conditions-III, the lifetime prevalence of MDD is 20.6% [2]. Serotonergic antidepressants take 6 to 8 weeks, on average, to produce clinical benefits and are ineffective in approximately two-thirds of patients with MDD [3]. In addition, serotonergic antidepressants have meaningful metabolic side effects, including weight gain, and cause sleep disruption and sexual dysfunction [4, 5]. Atypical antipsychotics are used as second-line adjunctive treatment; they are also marginally effective and have serious side effects [6]. There is an urgent medical need for a rapidly effective, safe, and well-tolerated treatment for MDD. Impaired neural plasticity caused by altered glutamatergic signaling has emerged as a mechanism of disease hypothesis for MDD, superseding the classic serotonergic hypothesis [7–10]. Neural plasticity is regulated by glutamatergic signaling via the N-methyl-D-aspartate receptor (NMDAR) [11, 12]. Uncompetitive NMDAR channel blockers reverse impaired neural plasticity and depressive-like behavior in animal models by restoring synaptic plasticity [13–18] and rapidly reverse MDD in patients [19–21].

Esmethadone (REL-1017) is the opioid-inactive dextro-isomer of methadone and a low affinity, low potency uncompetitive NMDAR antagonist [22, 23]. Esmethadone showed efficacy in animal models of depressive-like behavior [14, 24] acting via a brain-derived neurotrophic factor (BDNF)-dependent mechanism and interestingly esmethadone increased BDNF in humans [25]. Unlike more potent NMDAR antagonists, esmethadone does not produce Olney’s lesions or other evidence of damage to cortical neurons in rats [26]. In animal models, esmethadone has no meaningful opioid agonist effects [27–29]. In animal models predictive of human abuse potential, esmethadone did not cause physical dependence, withdrawal signs, or reinforcing effects [30, 31]. In human studies, esmethadone did not have meaningful opioid agonist effects and did not show meaningful abuse potential [32–34]. While esmethadone is not approved for any indication, it has been available to researchers since the 1940s. There have been no known cases of abuse with esmethadone. Esmethadone does not interconvert to levomethadone in vivo [35], and there is no known method for converting esmethadone to levomethadone in vitro.

Phase 1 and Phase 2 results with esmethadone provided safety, tolerability, and pharmacokinetic (PK) results across a range of doses sufficient to inform the design and conduct of the human abuse potential (HAP) studies [19, 35]. Phase 2 results with esmethadone showed rapid, robust, and sustained antidepressant effects at 25 and 50 mg oral daily doses and confirmed the favorable safety and tolerability profile seen in Phase 1 studies [19]. Phase 3 trials with esmethadone are ongoing and are expected to enroll approximately 1000 patients with MDD (ClinicalTrials.gov Identifiers: NCT04688164; NCT05081167; NCT04855747; NCT04855760). If the results of the esmethadone Phase 3 trials replicate Phase 2 results, esmethadone could offer a safe and well-tolerated rapid treatment for MDD. Although animal and human data indicate no meaningful opioid agonist effect and no ketamine-like dissociative effects with esmethadone, its potential use in a large population of patients with MDD, a patient population vulnerable to substance use disorder, warranted further evaluation. Therefore, a full HAP evaluation was conducted in two studies comparing esmethadone with oxycodone, an opioid with known abuse potential, and with ketamine, an NMDAR antagonist with known abuse potential. Racemic methadone and its isomers are currently Schedule II controlled substances in the United States and are also internationally controlled in Schedule I of the Single Convention on Narcotic Drugs, 1961. Chiral configuration is known to impart opioid activity to molecules: as a rule, for chiral molecules, only one of the two enantiomers is opioid active [7–9]. Dextromethorphan, an unscheduled, over the counter antitussive, is the opioid inactive dextro-isomer of racemetorphan, which is a schedule II narcotic, like the opioid active levo-isomer levomethorphan. While it is known that opioid receptor affinity and opioid agonist effects are stereoselective [36, 37], because of the structural similarity between esmethadone and levomethadone, an opioid agonist molecule, in the first study we compared esmethadone with oxycodone, which is a Schedule II opioid (Oxycodone Study). Because of the known NMDAR antagonist activity of esmethadone [22, 23], in the second study we compared esmethadone with ketamine, a Schedule III NMDAR antagonist (Ketamine Study). As an additional exploratory endpoint of the Ketamine Study, esmethadone was also compared to dextromethorphan (DXM), an unscheduled, over-the-counter NMDAR antagonist and antitussive medication. DXM in combination with quinidine is FDA approved for the treatment of pseudobulbar affect. DXM in combination with bupropion has shown efficacy for MDD [20, 38] and has been recently FDA approved for the treatment of MDD.

## Methods

These studies were conducted in accordance with relevant federal regulations of the Declaration of Helsinki, in compliance with the International Council for Harmonisation good clinical practice guidelines, and according to the appropriate regulatory requirements in the United States. Study protocols were reviewed and approved by a qualified Institutional Review Board. All participants signed the written informed consent prior to study procedures.

### Study designs

Each study utilized a single-dose, randomized, double-blind, active- and placebo-controlled crossover design to assess the abuse potential of esmethadone compared with oxycodone (Study 1—Oxycodone Study) or ketamine (Study 2—Ketamine Study) in healthy recreational drug users. All study drug administration and assessments were conducted in an inpatient setting. The overall design was consistent with FDA guidelines for assessing HAP [39]. Each study included a Screening Phase, a Qualification Phase, a Treatment Phase, and a Follow-up visit (Supplemental Figs. 1 and 2). Each Qualification Phase was conducted as a single-dose, randomized, crossover trial during which participants received single doses of oxycodone (40 mg, oral; Oxycodone Study) or ketamine (0.5 mg/kg, 40-min intravenous [IV] infusion; Ketamine Study), and placebo (oral matching placebo for the Oxycodone Study; IV and oral matching placebo for the Ketamine Study). To be eligible for the treatment phase of each study, participants had to tolerate the positive control and who could discriminate the positive control from placebo (i.e., Drug Liking bipolar visual analog scale (VAS) maximum effect (E_max_) of ≥65 points for the positive control and ≥15 point difference compared with placebo). Participants had to show an appropriate response following placebo administration, which was defined as scoring within the neutral range (i.e., 40 to 60 points on Drug Liking bipolar VAS) and also acceptable neutral responses on other scales. If a participants showed a positive response on Drug Liking following placebo (>60), they were excluded. In the Treatment Phase, eligible participants were randomized to receive each of the planned study drugs in a crossover manner. Study drug administration in each treatment period was separated by a minimum washout interval of 11 days. While there is always the potential for functional unblinding, double-and triple-dummy procedures and multiple treatment sequences were put in place to mitigate this risk. The ketamine study included two comparators, further decreasing the potential for functional unblinding.

### Participants

Participants were healthy individuals 18 to 55 years of age, inclusive, who were experienced with nontherapeutic (recreational) drug use. In the Oxycodone Study (Study 1), participants had prior experience with recreational opioid use (defined as ≥10 lifetime occasions of use and ≥1 use in the 12 weeks prior to Screening). In the Ketamine Study (Study 2), participants had prior experience with NMDAR antagonists (e.g., ketamine, esketamine, phencyclidine [PCP], DXM), and had specifically used ketamine ≥1 in their lifetime and had ≥1 use of drugs for nonmedical purposes by either the intranasal or IV route in the past year. Participants were recruited separately for each study and were compensated according to IRB-approved parameters. In addition, participants had a body mass index (BMI) ranging from 18 to 35 kg/m^2^, were healthy according to physical examination, medical history, vital signs, clinical laboratory assessments, and 12-lead electrocardiogram (ECG); and had negative urine drug screens and pregnancy tests (females) at each visit. Participants with a history/presence of drug or alcohol dependence or psychiatric disorder, according to the Diagnostic and Statistical Manual of Mental Disorders (4th edition, text revision) or who had ever participated in a substance or alcohol rehabilitation program were excluded. This was defined as any psychiatric disease that was anticipated in the Investigator’s or Medical Monitor’s opinion to be clinically significant and to potentially compromise safety or adversely affect the evaluation of the study data. No participant was excluded in either study for failing to satisfy this inclusion/exclusion criteria. Other exclusion criteria included corrected QT interval by Fridericia (QTcF) > 450 ms, pregnancy or breastfeeding (females), and allergy to NMDAR antagonists or related drugs or allergy to opioids. Concomitant medications (except acetaminophen, hormonal contraceptives, and hormone replacement therapy) were prohibited during the study.

### Study drugs and dose selection

Esmethadone doses of 25 mg, 75 mg (3x planned therapeutic dose and planned loading dose for the treatment of MDD, and 150 mg (6x therapeutic dose and the maximum tolerated dose [MTD]) [35], administered as 25 mg tablets (Patheon Pharma Services), were selected for evaluation in the Treatment Phase of both studies. The 25 mg and 75 mg doses were selected in accordance with FDA Guidance recommendations to evaluate the planned therapeutic daily dose, and a dose that is 2 to 3 times the planned therapeutic dose; the 75 mg dose is also the planned loading dose in patients. In consideration of its pharmacological class and that experienced drug users may exhibit a greater tolerance and seek higher doses, 150 mg was selected as it is 6 times the planned therapeutic dose.

Oxycodone, a Schedule II opioid, was selected as the positive control in the Oxycodone Study (Study 1). The dose of oxycodone (40 mg; administered as 20 mg over-encapsulated tablets) was consistent with those previously evaluated in HAP studies [40–49].

Ketamine, a Schedule III NMDAR antagonist, was selected as the positive control in the Ketamine Study (Study 2). The dose and infusion duration of IV ketamine (0.5 mg/kg over 40 min) was consistent with a previous HAP study (CDER review of NDA 211243 [esketamine]). DXM, an unscheduled, over-the-counter, NMDAR antagonist, and antitussive drug, was selected as an exploratory comparator in the Ketamine Study. The oral dose of DXM (300 mg capsule) was based on a prior single ascending dose study showing that this dose had detectable subjective effects but was not associated with prominent emesis that was more commonly observed at higher supratherapeutic doses of 400 to 800 mg [50].

To ensure blinding, study drugs were administered in a double- (Oxycodone Study) or triple- (Ketamine Study) dummy fashion, where participants received the same number of tablets and capsules (and a 40-min IV infusion in the Ketamine Study) administered as a combination of active or placebo product in each treatment period.

### Pharmacodynamic assessments

Bipolar 100-point VAS of “at-the-moment” Drug Liking, Overall Drug Liking, and Take Drug Again were used to measure the balance of positive and negative effects. The E_max_ of “at-the-moment” Drug Liking VAS was defined as the primary endpoint. Unipolar “at-the-moment” 100-point VAS were used to measure positive (High, Good Effects), negative (Bad Effects), and other (Any Effects, Alertness/Drowsiness) subjective drug effects. In the Ketamine Study, perceptual/dissociative effects were assessed using “at-the-moment” 100-point Hallucinations and Bowdle VAS (13-item scale rating current feelings) [51]. At-the-moment VAS was administered predose and 0.25, 0.5, 1, 1.5, 2, 3, 4, 5, 6, 7, 8, 10, 12, 24, 36, and 48 h post dose. Scales that referred specifically to drug effects were not administered predose. Overall Drug Liking VAS and Take Drug Again VAS were administered 12 and 24 h post dose. Drug Similarity VAS, administered at 12 h post dose, was used to estimate the class of drugs that the participants identified as being most similar to each of the treatments. Pupillometry was performed at predose and 0.5, 1, 2, 3, 4, 6, 8, 12, 24, 36, and 48 h post dose in the Oxycodone Study.

### Pharmacokinetic assessments

Blood samples were collected predose and 0.5, 1, 1.5, 2, 3, 4, 5, 6, 8, 12, 24, 36, and 48 h post dose. In the Oxycodone Study, plasma samples were analyzed for REL-101 and oxycodone. In the Ketamine Study, plasma samples were analyzed for esmethadone, ketamine, norketamine, DXM, and dextrorphan.

### Safety assessments

Safety assessments were performed throughout all phases of the studies and included adverse events (AE; spontaneous participant reports), vital signs (blood pressure, pulse rate, oxygen saturation, and respiratory rate), 12-lead ECGs, continuous cardiac telemetry, pulse oximetry (from ≥15 min predose to ≥4 h post dose), clinical laboratory testing, physical examinations, and the Columbia-Suicide Severity Rating Scale (C-SSRS) [52].

### Statistical analyses

Pharmacodynamic (PD) analyses were conducted using the Completer Population, defined as participants who completed all treatment periods and had at least one response on the VAS for Drug Liking within 2 h of peak plasma concentrations (T_max_) for each treatment [39]. For both studies, PD analyses were also performed for the Modified Completer Population, which excluded participants who had similar Drug Liking E_max_ scores (within 5 points difference) across all study treatments (including placebo) and excluded participants with an E_max_ for placebo >60 and a difference between E_max_ for oxycodone and placebo of ≤5.

The primary PD endpoint, Drug Liking VAS E_max_, was analyzed using a 1-sided hypothesis test at a significance level of α = 0.05 and reported with 95% confidence intervals (CIs) and prespecified margins for each of the hypotheses. For the validation test (positive control vs. placebo), the margin of 15 was selected based on prior studies (CDER review of NDA 211243 [esketamine]) [44, 53–55]. For the test between the positive control and esmethadone (abuse potential relative to oxycodone or ketamine), a margin of 0 was applied. A margin of 11 was selected for the statistical test between esmethadone and placebo and between DXM and placebo (abuse potential relative to placebo) for the primary endpoint, based on a meta-analysis of 8 HAP studies [56]. The tests were conducted sequentially and thus, no multiplicity adjustment was needed. No specific margins were prespecified for the inferential analysis of secondary endpoints because there is currently no supporting literature that can aid in identification of a margin. Therefore, secondary endpoint results are presented as descriptive statistics.

Pharmacodynamic endpoints (E_max_), area under/over the effect curve) were initially analyzed using a linear mixed effects model containing treatment, period, sequence, and first-order carryover effect as fixed effects (SAS version 9.4 or higher, SAS Institute Inc., Cary, NC, USA). The participant nested-within-treatment sequence was included as a random effect. Baseline was also included as a covariate where applicable (i.e., for measures evaluated predose). The first-order carryover effect was the previous treatment received in the Treatment Phase. If the carryover effect was found to be nonsignificant at the 25% level, then the term was dropped from the model [57]. If the carryover effect was significant at the 25% level, but not at the 5% level, then the carryover effect term was retained in the model; if the carryover effect was significant at the 5% level, a first-period analysis was conducted. The residuals from the mixed effects model were investigated for normality using the Shapiro–Wilk W test [58]. Parameters were analyzed under the assumption of a normal distribution of errors if the *p* value of the test was ≥0.01. If the *p* value was <0.01 for the Shapiro–Wilk W test on the residuals from the mixed model, a test of skewness was conducted on each paired difference. If the distribution of the paired differences was not skewed (−0.5 < skewness value < 0.5), then the endpoint was analyzed using a paired t-test. If the distribution of the paired differences was skewed (skewness value ≤ 0.5 or skewness value > 0.5), then the endpoint was analyzed nonparametrically using the Sign Test. Based on assumptions, derived from prior studies [54] and CDER review of NDA 211243 [esketamine]). that the true mean difference between the active comparators and placebo is approximately 35 points, the estimated sample size of 43 completer subjects provided greater than 90% power.

Pharmacokinetic (PK) parameters (peak plasma concentration [C_max_], T_max_, area under the concentration-time curve from 0 to last measurable concentration [AUC_0-last_]) for each analyte were calculated using non-compartmental analysis (Phoenix WinNonlin, version 8.1, Certara, L.P., Princeton, NJ, USA) for the PK Population, which included all participants who received at least 1 dose of active study drug and had at least 1 measurable PK sample for the respective treatment. Derived parameters were summarized descriptively. All safety analyses were summarized descriptively using the Safety Population, which included all participants who received at least 1 dose of study drug in the Treatment Phase.

## Results

### Participant disposition and demographics

In the Oxycodone Study (Study 1), 50 participants were randomized at two clinical sites (Hassman Research Institute [*n* = 8] and Ohio Clinical Trials [*n* = 42]) to the Treatment Phase, and 47 completed all treatment periods and were included in the Completer Population. Six participants discontinued early: 5 were lost to follow-up (3 of these 5 subjects completed all 5 treatment periods but did not attend the final follow-up visit and were included in the Completer Population), and 1 was discontinued due to noncompliance or major protocol violation (repeated visit cancellation/no show). In the Ketamine Study (Study 2), 54 participants were randomized at two clinical sites (Ohio Clinical Trials [*n* = 32] and Woodland Research Northwest [*n* = 22]) to the Treatment Phase, and 51 completed all treatment periods and were included in the Completer Population. Three participants discontinued early: 1 withdrew consent, 1 discontinued for safety reasons (AEs of elevated alanine aminotransferase, aspartate aminotransferase, and blood lactate dehydrogenase at admission to treatment period 6 [last treatment: DXM 300 mg]), and 1 was discontinued for administrative reasons.

In the Oxycodone Study, most participants were male, Black or African-American and non-Hispanic, with a mean age of 36.3 years (Table 1). All participants reported prior experience with opioids, and the majority reported a history of cannabinoid use. Recreational use of depressants also was relatively common, whereas few participants reported recreational use of stimulants, hallucinogens, or dissociative anesthetics. In the Ketamine Study, most participants were male, white, and non-Hispanic, with a mean age of 34.4 years (Table 1). All participants reported prior experience with dissociative anesthetics, and the majority reported a history of cannabinoids use, hallucinogen use, and stimulant use. Recreational use of opioids also was relatively common, whereas few participants reported recreational use of depressants or nitrite inhalants.

**Table 1:** Baseline characteristics—Oxycodone Study.

	Oxycodone Study		Ketamine Study	
Demographic variable	Safety population *N* = 50	Completer population *N* = 47	Safety population *N* = 54	Completer population *N* = 51
Age, years^a^	36.3 ± 8.9	36.3 ± 9.1	34.4 ± 9.8	34.4 ± 9.8
Male, *n* (%)	40 (80.0)	37 (78.7)	37 (68.5)	35 (68.6)
Race, *n* (%)				
White	21 (42.0)	20 (42.6)	34 (63.0)	32 (62.7)
Black	28 (56.0)	27 (57.4)	15 (27.8)	14 (27.5)
American Indian or Alaskan Native	1 (2.0)	0	2 (3.7)	2 (3.9)
Asian	0	0	2 (3.7)	2 (3.9)
Other	0	0	2 (3.7)	2 (3.9)
Hispanic or Latino, *n* (%)	6 (12.0)	5 (10.6)	4 (7.4)	4 (7.8)
Body mass index, kg/m^2 a^	26.8 ± 4.2	26.8 ± 4.2	25.2 ± 3.8	25.2 ± 3.7

^a^Mean ± standard deviation.

### Pharmacodynamics

#### Oxycodone Study

**Fig. 1:**
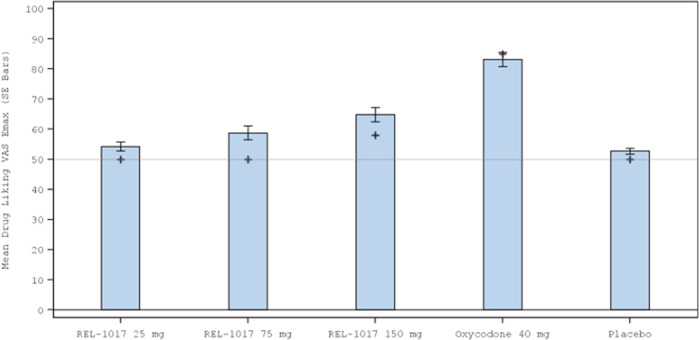
Mean (standard error) Drug Liking VAS E_max_ by Treatment During the Treatment Phase for the Oxycodone Study (Completer Population). + = median value; *p* < 0.001 for median difference between oxycodone and placebo.

Effects on Drug Liking VAS E_max_ (primary study endpoint) are shown in Fig. 1. The validity of the study was determined from the comparison of Drug Liking VAS E_max_ between the positive control, oxycodone 40 mg, and placebo. The median (Q1, Q3) difference was 35.0 (18.0, 49.0; *p* < 0.001) for the Completer Population, indicating that oxycodone had a meaningful and statistically significantly higher Drug Liking VAS E_max_ compared with placebo, using a prespecified margin of 15 (Tables 2 and 3). The abuse potential of esmethadone relative to oxycodone was determined from the comparison of Drug Liking VAS E_max_ of each dose with the positive control. Drug Liking VAS E_max_ for each esmethadone dose was meaningfully and significantly lower than oxycodone at a prespecified margin of 0 (mean/median difference between oxycodone and each dose of esmethadone ≥19; all *p* < 0.001) (Tables 2 and 3). The abuse potential of esmethadone relative to placebo was determined from the comparisons of Drug Liking VAS E_max_ of each dose with placebo. The median difference from placebo was 0 for esmethadone 25 mg and 75 mg (*p* < 0.001) and 7.0 for the 150 mg dose (*p* = 0.036), indicating that esmethadone was not meaningfully different from placebo and was statistically equivalent to placebo at doses up to 6 times the planned therapeutic dose and MTD (Tables 2 and 3). Mean Drug Liking VAS scores were maintained close to placebo scores over 24 h (Supplemental Figure 3).

**Table 2:** Drug Liking bipolar VAS E_max_—Oxycodone Study.

	Oxycodone Study				
Statistic	Esmethadone 25 mg (*N* = 47)	Esmethadone 75 mg (*N* = 47)	Esmethadone 150 mg (*N* = 47)	Oxycodone 40 mg (*N* = 47)	Placebo (*N* = 47)
Mean (SD)	54.2 (10.35)	58.7 (15.82)	64.9 (16.58)	83.2 (16.57)	52.7 (6.52)
Median	50.0	50.0	58.0	85.0	50.0
Range	50–100	50–100	50–100	50–100	50–80

Drug Liking VAS is a bipolar scale where a score of 0 represents “strong disliking,” a score of 100 represents “strong liking,” and a score of 50 represents “neither like nor dislike” (neutral point). The question text is, “At this moment, my liking for this drug is?”.

*E*_*max*_ maximum effect, *range* minimum–maximum, *SD* standard deviation, *VAS* visual analog scale.

**Table 3:** Inferential analysis of Drug Liking VAS E_max_—esmethadone vs. oxycodone.

Pairwise comparisons	Mean/median of intra-participant difference	95% CI/Quartiles	*P*-value^a^
*Study validity*			
Oxycodone 40 mg – Placebo	35.0	(18.0, 49.0)^b^	**<0.001**
*Drug Liking VAS E*_*max*_ *relative to oxycodone*			
Oxycodone 40 mg – Esmethadone 25 mg	34.0	(19.0, 48.0)^b^	**<0.001**
Oxycodone 40 mg – Esmethadone 75 mg	25.0	(12.0, 41.0)^b^	**<0.001**
Oxycodone 40 mg – Esmethadone 150 mg	19.0	(5.0, 34.0)^b^	**<0.001**
*Drug Liking VAS E*_*max*_ *relative to placebo*			
Esmethadone 25 mg – Placebo	0.0	(0.0, 1.0)^b^	**<0.001**
Esmethadone 75 mg – Placebo	0.0	(−1.0, 8.0)^b^	**<0.001**
Esmethadone 150 mg – Placebo	7.0	(0.0, 23.0)^b^	**0.036**

Note: Friedman’s test was used to assess overall treatment effects: *p*-value < 0.001 for both populations.

Study validity hypothesis (#1): H_o_: µ_C_ – µ_P_ ≤ 15 vs. H_a_: µ_C_ – µ_P_ > 15; 1-sided test (α = 0.05).

Abuse potential relative to oxycodone hypothesis (#2): H_o_: µ_C_ – µ_T_ ≤ 0 vs. H_a_: µ_C_ – µ_T_ > 0; 1-sided test (α = 0.05).

Abuse potential relative to placebo hypothesis (#3): H_o_: µ_T_ – µ_P_ ≥ 11 vs. H_a_: µ_T_ – µ_P_ < 11; 1-sided test (α = 0.05) where P = placebo; C = positive control; and T = test drug. In this equivalence test, a significant *p*-value (<0.05) indicates the response to REL-1017 was statistically equivalent to that of placebo.

Bolded *p*-values are statistically significant. A statistically significant *p*-value for the comparison of esmethadone vs. placebo indicates that esmethadone at that dose level has a response profile equivalent to placebo.

*CI* confidence interval, *E*_*max*_ maximum effect, *VAS* visual analog scale.

^a^A paired *t* test was used to assess the mean difference between the 2 treatments; mean and 95% CI are presented.

^b^The Sign test was used to assess the median difference between the 2 treatments; median and quartiles are presented.

Consistent with the primary endpoint, oxycodone had greater effects compared with placebo on all secondary endpoints, including global effects (Overall Drug Liking, Take Drug Again VAS), positive effects (Good Effects, High VAS), and other effects (Any Effects, Alertness/Drowsiness VAS) and all doses of esmethadone had lower effects on all secondary endpoints compared with oxycodone (Table 4). Based on Drug Similarity VAS, participants rated oxycodone to be most similar to the category of Opioids (mean/median scores of 81.6 and 100, respectively). Participants did not perceive esmethadone 25 mg or 75 mg as similar to Opioids, and esmethadone 150 mg was rated as only modestly similar to Opioids, with a mean score of 38.8 and median score of 26 on unipolar 0-100 VAS (Table 4). Mean (SD) maximum pupillary constriction (MPC) values were was 0.847 (0.5372); 1.312 (0.5600); 2.114 (0.7629) mm for esmethadone 25 mg; esmethadone 75 mg; esmethadone 150 mg, respectively; 3.036 (1.0272) mm for oxycodone 40 mg; and 0.685 (0.5153) mm for placebo. Mean values were significantly lower for each esmethadone dose vs. oxycodone (*p* < 0.001).

**Table 4:** Descriptive statistics for secondary endpoints—esmethadone vs. oxycodone.

Oxycodone Study					
Statistic	Esmethadone 25 mg (*N* = 47)	Esmethadone 75 mg (*N* = 47)	Esmethadone 150 mg (*N* = 47)	Oxycodone 40 mg (*N* = 47)	Placebo (*N* = 47)
*Overall Drug* Liking bipolar VAS					
Mean (SD)	53.1 (9.20)	58.1 (19.33)	61.4 (18.67)	73.9 (24.02)	52.6 (12.50)
Median	50.0	50.0	51.0	73.0	50.0
Range	36–85	12–100	16–100	9–100	0–98
*Take Drug Again* bipolar VAS					
Mean (SD)	52.6 (17.34)	57.8 (24.33)	61.2 (22.94)	76.1 (26.97)	51.1 (16.66)
Median	50.0	50.0	50.0	83.0	50.0
Range	0–100	0–100	0–100	7–100	0–100
*High* unipolar VAS					
E_max_					
Mean (SD)	9.9 (22.65)	21.4 (31.88)	31.3 (34.66)	74.8 (26.74)	7.2 (17.33)
Median	0.0	1.0	17.0	83.0	0.0
Range	0–100	0–100	0–100	5–100	0–77
*Good Effects* unipolar VAS					
E_max_					
Mean (SD)	9.9 (23.55)	22.3 (32.75)	32.9 (36.07)	73.1 (26.26)	9.1 (21.67)
Median	0.0	0.0	19.0	79.0	0.0
Range	0–100	0–100	0–100	7–100	0–96
*Bad Effects* unipolar VAS E_max_					
E_max_					
Mean (SD)	3.3 (11.81)	7.4 (21.32)	12.7 (24.77)	27.4 (30.37)	1.1 (4.79)
Median	0.0	0.0	0.0	14.0	0.0
Range	0–72	0–100	0–89	0–100	0–28
*Alertness/Drowsiness* unipolar VAS					
E_min_					
Mean (SD)	44.4 (10.44)	41.2 (14.77)	34.1 (16.27)	18.4 (14.94)	45.9 (12.16)
Median	50.0	50.0	37.0	16.0	50.0
Range	0–50	0–50	0–54	0–50	0–90
*Any Effects* unipolar VAS					
E_max_					
Mean (SD)	10.9 (24.66)	25.9 (34.37)	37.1 (36.45)	78.1 (26.28)	7.3 (17.34)
Median	0.0	4.0	28.0	85.0	0.0
Range	0–100	0–100	0–100	5–100	0–72
*Drug Similarity* unipolar VAS Scores at 12 h:					
*Opioids*					
*n*	46	44	47	47	47
Mean (SD)	9.8 (26.11)	17.7 (30.76)	38.8 (40.43)	81.6 (30.84)	9.8 (23.19)
Median	0.0	0.0	26.0	100.0	0.0
Range	0–100	0–100	0–100	0–100	0–100

Overall Drug Liking VAS is a bipolar scale where a score of 0 represents “strong disliking,” a score of 100 represents “strong liking,” and a score of 50 represents “neither like nor dislike” (neutral point). The question text is, “Overall, my liking for this drug is.”

Take Drug Again VAS is a bipolar scale where a score of 0 represents “definitely not,” a score of 100 represents “definitely so,” and a score of 50 represents “neutral” (neutral point). The question text is, “I would take this drug again.”

*E*_*max*_ maximum effect, *range* minimum–maximum, *SD* standard deviation, *VAS* visual analog scale.

#### Ketamine Study

**Fig. 2:**
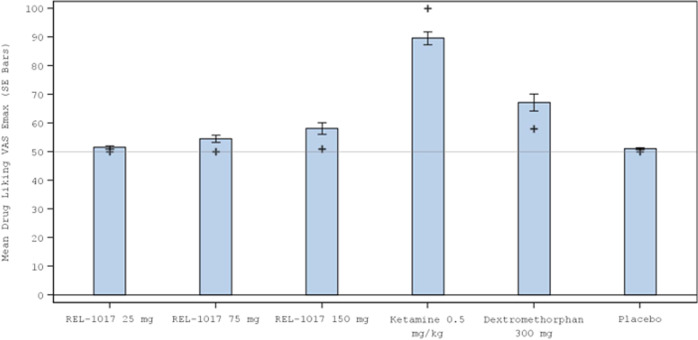
Mean (standard error) Drug Liking VAS E_max_ by Treatment During the Treatment Phase for the Ketamine Study (Completer Population). + = median value; *p* < 0.001 for median difference between ketamine and placebo.

Effects on Drug Liking VAS E_max_ (primary endpoint) are shown in Fig. 2. The validity of the study was determined from the comparison of Drug Liking VAS E_max_ between the positive control, ketamine 0.5 mg/kg administered IV over 40 min, and placebo (Table 5). The median (Q1, Q3) difference was 49.0 (27.0, 50.0; *p* < 0.001) for the Completer Population, indicating that ketamine had a meaningful and statistically significantly higher Drug Liking VAS E_max_ compared with placebo, using a prespecified margin of 15. The abuse potential of esmethadone relative to ketamine was determined from the comparison of Drug Liking VAS E_max_ of each esmethadone dose with ketamine. Drug Liking VAS E_max_ for each esmethadone dose was meaningfully and statistically significantly lower than that of ketamine at a prespecified margin of 0 (mean/median difference between ketamine and each dose of esmethadone ≥34.0; all *p* < 0.001) (Table 6). The abuse potential of esmethadone compared to placebo was determined from the comparisons of Drug Liking VAS E_max_ of each dose with placebo. The median difference from placebo was 0 for all tested doses of esmethadone (*p* ≤ 0.003), indicating that esmethadone was equivalent to placebo at doses up to 6 times the planned therapeutic dose. In the exploratory comparisons with DXM, Drug Liking VAS E_max_ for DXM 300 mg was meaningfully and significantly lower than the Drug Liking VAS E_max_ for ketamine (mean difference: 21.6; *p* < 0.001). DXM was found not to be equivalent to placebo in Drug Liking VAS E_max_ using the margin of 11 (*p* = 0.39 (Table 6). Drug Liking VAS E_max_ for all tested doses of esmethadone were significantly lower compared to DXM (mean/median differences ≥8.0; *p* ≤ 0.002) (Table 6). Mean Drug Liking VAS scores were maintained close to placebo scores over 24 h (Supplemental Figure 3).

**Table 5:** Drug Liking bipolar VAS E_max_—Ketamine Study.

	Ketamine Study					
Statistic	Esmethadone 25 mg (*N* = 51)	Esmethadone 75 mg (*N* = 51)	Esmethadone 150 mg (*N* = 51)	Ketamine 0.5 mg/kg (*N* = 51)	DXM 300 mg (*N* = 51)	Placebo (*N* = 51)
Mean (SD)	51.4 (3.28)	54.9 (9.58)	59.2 (14.38)	90.0 (14.52)	68.4 (18.39)	50.9 (2.23)
Median	50.0	50.0	51.0	100.0	60.0	50.0
Range	50–66	50–100	50–100	50–100	50–100	50–63

Drug Liking VAS is a bipolar scale where a score of 0 represents “strong disliking,” a score of 100 represents “strong liking,” and a score of 50 represents “neither like nor dislike” (neutral point). The question text is, “At this moment, my liking for this drug is?”.

*E*_*max*_ maximum effect, *range* minimum–maximum, *SD* standard deviation, *VAS* visual analog scale. Esmethadone and DXM were oral administration; ketamine IV administration.

**Table 6:** Inferential analysis of Drug Liking VAS E_max_—esmethadone vs. ketamine and DMX.

*Study validity*			
Ketamine 0.5 mg/kg – Placebo	49.0	(27.0, 50.0)^b^	**<0.001**
*Drug Liking VAS E*_*max*_ *relative to ketamine*			
Ketamine 0.5 mg/kg – Esmethadone 25 mg	48.0	(27.0, 50.0)^b^	**<0.001**
Ketamine 0.5 mg/kg – Esmethadone 75 mg	40.0	(25.0, 50.0)^b^	**<0.001**
Ketamine 0.5 mg/kg – Esmethadone 150 mg	34.0	(18.0, 49.0)^b^	**<0.001**
*Drug Liking VAS E*_*max*_ *relative to placebo*			
Esmethadone 25 mg – Placebo	0.0	(0.0, 0.0)^b^	**<0.001**
Esmethadone 75 mg – Placebo	0.0	(0.0, 3.0)^b^	**<0.001**
Esmethadone 150 mg – Placebo	0.0	(0.0, 14.0)^b^	**0.003**
*Exploratory comparisons*			
DXM 300 mg – Placebo	8.0	(0.0, 35.0)^b^	0.39
DXM 300 mg – Esmethadone 25 mg	10.0	(0.0, 34.0)^b^	**<0.001**
DXM 300 mg – Esmethadone 75 mg	13.5	(8.6, ∞)^a^	**<0.001**
DXM 300 mg – Esmethadone 150 mg	9.2	(4.1, ∞)^a^	**0.002**
Ketamine 0.5 mg/kg – DXM 300 mg	21.6	(17.1, ∞)^a^	**<0.001**

Note: Friedman’s test was used to assess overall treatment effects: *p* value < 0.001 for both populations.

Study validity hypothesis (#1): H_o_: µ_C_ – µ_P_ ≤ 15 vs. H_a_: µ_C_ – µ_P_ > 15; 1-sided test (α = 0.05).

Abuse potential relative to ketamine hypothesis (#2): H_o_: µ_C_ – µ_T_ ≤ 0 vs. H_a_: µ_C_ – µ_T_ > 0; 1-sided test (α = 0.05).

Abuse potential relative to placebo hypothesis (#3): H_o_: µ_T_ – µ_P_ ≥ 11 vs. H_a_: µ_T_ – µ_P_ < 11; 1-sided test (α = 0.05) where P = placebo; C = positive control; and T = test drug.

Exploratory hypotheses:

H_o_: μ_C2_ – μ_P_ ≤ 0 vs. H_a_: μ_C2_ – μ_P_ > 0; 1-sided test (α = 0.05).

H_o_: μ_C2_ – μ_T_ ≤ 0 vs. H_a_: μ_C2_ – μ_T_ > 0; 1-sided test (α = 0.05).

H_o_: μ_C1_ – μ_C2_ ≤ 0 vs. H_a_: μ_C1_ – μ_C2_ > 0; 1-sided test (α = 0.05), where P = placebo; C_1_ = positive control (ketamine); C_2_ = exploratory comparator (DXM) and T = test drug.

*CI* confidence interval, *E*_*max*_ maximum effect, *VAS* visual analog scale.

^a^A paired *t* test was used to assess the mean difference between the 2 treatments; mean and 95% CI are presented.

^b^The Sign test was used to assess the median difference between the 2 treatments; median and quartiles are presented.

Bolded *p*-values are statistically significant. A statistically significant *p*-value for the comparison of esmethadone vs. placebo indicates that esmethadone at that dose level has a response profile equivalent to placebo. The nonsignificant *p*-value reported for DXM vs. placebo indicates that DXM at that dose level lacks placebo equivalency.

Ketamine had greater effects compared with placebo on all secondary endpoints (Table 7). All doses of esmethadone showed lower effects on all endpoints compared with ketamine, including Overall Drug Liking VAS, Take Drug Again VAS, Good Effects VAS, High VAS, and other effects including perceptual effects. In contrast with ketamine and DXM, esmethadone did not cause hallucinations or perceptual effects. Mean (SD) unipolar E_max_ scores for Hallucinations VAS were 0.2 (0.55); 0.3 (0.77); 0.6 (0.24) points for esmethadone 25 mg; esmethadone 75 mg; esmethadone 150 mg, respectively; 23.1 (38.5) points for ketamine; 7.5 (19.21) points for DXM; and 0.2 (0.45) points for placebo. Based on Drug Similarity VAS, ketamine was rated to be most similar to the category of Ketamine (mean/median scores of 93.1 and 100, respectively) and less so with Opioids (22.8 and 4.0, respectively). Participants did not perceive esmethadone as similar to Ketamine or Opioids, with mean scores <20 and median scores of 0.

**Table 7:** Descriptive statistics for secondary endpoints—esmethadone vs. ketamine and DMX.

Ketamine Study						
Statistic	Esmethadone 25 mg (*N* = 51)	Esmethadone 75 mg (*N* = 51)	Esmethadone 150 mg (*N* = 51)	Ketamine 0.5 mg/kg (*N* = 51)	DXM 300 mg (*N* = 51)	Placebo (*N* = 51)
*Overall Drug* Liking bipolar VAS						
Mean (SD)	51.3 (7.98)	50.8 (13.72)	52.9 (20.13)	87.4 (19.35)	57.7 (30.82)	47.7 (9.68)
Median	50.0	50.0	50.0	100.0	59.0	50.0
Range	43–100	0–00	0–100	41–100	0–100	0–54
*Take Drug Again* bipolar VAS						
Mean (SD)	50.5 (10.87)	50.0 (18.25)	53.5 (24.40)	88.2 (21.95)	55.2 (32.44)	48.8 (13.29)
Median	50.0	50.0	50.0	100.0	51.0	50.0
Range	0–100	0–100	0–100	1–100	0–100	0–100
*High* unipolar VAS						
E_max_						
Mean (SD)	2.9 (6.44)	10.2 (18.94)	17.3 (25.92)	87.7 (21.70)	60.8 (36.68)	2.1 (4.26)
Median	0.0	0.0	4.0	100.0	73.0	0.0
Range	0–27	0–79	0–100	19–100	1–100	0–19
*Good Effects* unipolar VAS						
E_max_						
Mean (SD)	2.9 (8.73)	10.2 (20.36)	19.5 (28.59)	86.3 (22.26)	47.2 (35.56)	2.7 (8.63)
Median	0.0	0.0	5.0	100.0	49.0	0.0
Range	0–50	0–100	0–100	14–100	0–100	0–57
*Bad Effects* unipolar VAS E_max_						
E_max_						
Mean (SD)	2.6 (11.62)	5.2 (14.55)	7.1 (18.20)	14.0 (28.36)	34.7 (37.47)	3.3 (9.19)
Median	0.0	0.0	0.0	0.0	22.0	0.0
Range	0–80	0–71	0–90	0–100	0–100	0–50
*Alertness/Drowsiness* unipolar VAS						
E_max_						
Mean (SD)	51.4 (7.03)	52.6 (9.51)	53.6 (10.00)	69.0 (21.29)	58.9 (15.82)	51.2 (6.99)
Median	50.0	50.0	50.0	54.0	50.0	50.0
Range	50–100	50–100	50–100	50–100	50–100	50–100
*Any Effects* unipolar VAS						
E_max_						
Mean (SD)	4.3 (11.56)	13.5 (21.48)	21.4 (28.47)	90.1 (20.11)	66.8 (35.71)	4.3 (8.03)
Median	0.0	2.0	8.0	100.0	79.0	1.0
Range	0–73	0–89	0–100	11–100	1–100	0–36
*Hallucinations* unipolar VAS						
E_max_						
Mean (SD)	0.2 (0.55)	0.3 (0.77)	0.6 (2.24)	23.1 (38.05)	7.5 (19.21)	0.2 (0.45)
Median	0.0	0.0	0.0	0.0	0.0	0.0
Range	0–3	0–5	0–14	0–100	0–100	0–2
*Bowdle—External Perception* unipolar VAS						
E_max_						
Mean (SD)	0.2 (0.58)	1.1 (3.21)	1.9 (4.56)	33.9 (31.61)	11.1 (17.83)	0.2 (0.63)
Median	0.0	0.0	0.2	21.3	2.3	0.0
Range	0–4	0–18	0–21	0–100	0–100	0–4
*Bowdle—Internal Perception* unipolar VAS						
E_max_						
Mean (SD)	0.1 (0.17)	0.4 (1.04)	0.3 (0.59)	17.5 (24.26)	6.9 (10.64)	0.3 (1.35)
Median	0.0	0.0	0.0	9.6	1.0	0.0
Range	0–1	0–5	0–3	0–100	0–40	0–10
*Drug Similarity at 12* *h* unipolar VAS						
Ketamine						
*n*	51	51	51	50	51	51
Mean (SD)	0.5 (2.34)	5.5 (15.26)	10.1 (24.20)	93.1 (21.64)	37.9 (36.35)	1.2 (6.79)
Median	0.0	0.0	0.0	100.0	34.0	0.0
Range	0–14	0–85	0–100	0–100	0–100	0–48
Opioids						
*n*	30	30	30	29	30	30
Mean (SD)	5.8 (19.06)	11.7 (28.54)	16.7 (29.03)	22.8 (34.35)	23.8 (33.72)	5.5 (19.59)
Median	0.0	0.0	0.0	4.0	0.0	0.0
Range	0–100	0–98	0–100	0–100	0–100	0–100

Overall Drug Liking VAS is a bipolar scale where a score of 0 represents “strong disliking,” a score of 100 represents “strong liking,” and a score of 50 represents “neither like nor dislike” (neutral point). The question text is, “Overall, my liking for this drug is.”

Take Drug Again VAS is a bipolar scale where a score of 0 represents “definitely not,” a score of 100 represents “definitely so,” and a score of 50 represents “neutral” (neutral point). The question text is, “I would take this drug again.”

*E*_*max*_ maximum effect; *range* minimum–maximum; *SD* standard deviation; *VAS* visual analog scale.

Pharmacodynamic analyses were also performed for the Modified Completer Population. The results for the Modified Completer Population for the Oxycodone Study and for the Ketamine Study are presented in Supplemental Tables 1 to 6. It should be noted that the Modified Completer Population excludes placebo responders; therefore, descriptive and inferential statistical comparisons of the test drug against placebo in the Modified Completer Population are biased towards showing lack of equivalency with placebo (placebo responders are eliminated but test drug responders are not). Inferential statistical comparisons of the test drug against the positive control in the Modified Completer Population may also be biased towards showing a greater difference between positive control and test drug by eliminating participants with low scores for the comparator drug. Whereas the performance of analyses on the Completer Population is established and accepted [39], the performance of additional analyses on the Modified Completer Population is evolving. The intent of making this analysis available is to stimulate interest in population enrichment strategies that may enhance the interpretation of HAP studies.

### Pharmacokinetics

In both studies, esmethadone geometric mean plasma exposures (C_max_ and AUC_0-last_) increased with increasing dose of esmethadone. Median T_max_ ranged from approximately 2 to 3 h post dose for all esmethadone doses in both studies, whereas T_max_ for oxycodone occurred at approximately 1 h post dose (Supplemental Figure 4 and Supplemental Table 7). The T_max_ for ketamine occurred at approximately 1 h post dose, the first post infusion timepoint, and T_max_ for norketamine was observed at 1.5 h post dose. Exposure to DXM and dextrorphan was highly variable, with a median T_max_ of 3 and 2 h, respectively (Supplemental Figure 5).

### Safety

#### Oxycodone Study

Overall, the highest incidence (≥5% participants at any dose) of treatment-emergent AEs (TEAEs) was observed with oxycodone 40 mg (52.1%), followed by esmethadone 150 mg (28.6%) (Supplemental Table 8). The incidence of TEAEs for placebo, esmethadone 25 mg, and esmethadone 75 mg was 12.2%, 12.8%, and 12.2%, respectively. The most common TEAEs with oxycodone were nausea, somnolence, pruritus, vomiting, dizziness, and hot flush. The most common AEs with esmethadone were nausea, headache, somnolence, and vomiting. The incidence of nausea, vomiting, and somnolence appeared to increase with the esmethadone dose; however, the incidence was lower compared with oxycodone. There were no reports of dizziness or hot flush with esmethadone. There were no deaths or serious AEs. No notable treatment-related changes or trends were observed for clinical laboratory, vital signs, ECG or C-SSRS results following esmethadone administration. Notably, there were no TEAEs related to QTc prolongation. Drug-induced QTcF prolongation was modest and was slightly higher for oxycodone compared to each dose of esmethadone, including the 150 mg dose (Supplemental Table 7). Overall, esmethadone was well-tolerated at doses up to 150 mg.

#### Ketamine Study

The highest incidence of TEAEs was observed with DXM (74.1%), while a similar incidence of TEAEs occurred with esmethadone 75 mg (41.5%) and 150 mg (39.5%) (Supplemental Table 8). The incidence of TEAEs with esmethadone 25 mg and ketamine (24.5% and 25.0%, respectively) was slightly lower compared to placebo (30.8%). The most common TEAEs with ketamine were headache and somnolence. The most common TEAEs with esmethadone were nausea, headache, and somnolence. The incidence of nausea appeared to increase with the esmethadone dose. DXM was associated with the highest incidence of nausea and vomiting; headache, somnolence, and pruritus also were common. There were no deaths or serious AEs. No TEAEs related to QTc prolongation occurred (Supplemental Table 8). No notable treatment-related changes or trends in clinical laboratory findings, vital signs or ECG results were observed following esmethadone administration. Overall, esmethadone was well-tolerated at doses up to 150 mg.

## Discussion

Esmethadone is a low potency uncompetitive NMDAR antagonist [22, 23] and promising rapid antidepressant candidate. There is experimental evidence that esmethadone may have opioid antagonistic activity to the agonist effects of levomethadone [59] and there is clinical evidence that esmethadone may have weak antagonistic activity on the respiratory depressant [60] and on subjective opioid effects [61] induced by levomethadone. Esmethadone is not yet approved for any indication and is currently a Schedule II controlled substances in the United States. If esmethadone is approved for the treatment of MDD, a rescheduling decision will be sought.

HAP studies provide important data for predicting recreational use in the community and provide information for labeling and for drug scheduling under the Controlled Substances Act (CSA). In both HAP studies, all three esmethadone doses (25 mg [therapeutic], 75 mg [3x therapeutic], and 150 mg [6x therapeutic and MTD]) had meaningful and statistically significant lower scores compared with positive control (oxycodone or ketamine) on the primary endpoint of Drug Liking VAS E_max_, and a similar pattern was observed for all secondary endpoints. Furthermore, Drug Liking VAS E_max_ for all doses of esmethadone were statistically equivalent to placebo, including the MTD, 150 mg, in both studies, using the Completer Population. The modest dose-effect relationship observed in both studies, which was confined to the range of placebo effects [56], indicates that there would be no meaningful incentive for drug abusers to increase the dose of esmethadone to achieve a greater effect. Pupillary constriction was less with esmethadone relative to oxycodone. Pupillary constriction with esmethadone in this study was similar to pupillary constriction induced by esmethadone in prior studies [35]. While pupillary constriction is a well-known opioid agonist effect, it is not always associated with other clinically meaningful opioid agonist effects. In fact, naloxone, a well-known opioid antagonist with no described opioid agonist effects, may cause pupillary constriction [62].

We presented results for the Completer Population as indicated in the FDA 2017 Guidance [39] (Tables 3 to 6) and for the Modified Completer Population (Supplemental Tables 1–6), as suggested by recent research trends in methodology for HAP studies. For the two studies presented here, the application of Modified Completer Population parameters did not meaningfully change the results and conclusions obtained with the Completer Population.

In summary, the results of these two, randomized, double-blind, active- and placebo-controlled, crossover studies testing the abuse potential of esmethadone in recreational drug users are consistent with the profile of a drug without meaningful abuse potential and are similar to results observed for unscheduled drugs without abuse potential such as eslicarbazepine and difelikefalin [42, 63] and Schedule V drugs such as lacosamide [64]. Furthermore, the Ketamine Study, in addition to showing meaningful and statistically significant lower abuse potential compared to ketamine, also showed statistically significant lower effects compared to DXM (Table 6), an unscheduled and over-the-counter NMDAR antagonist and antitussive medication. Mean values for Drug Liking tended to be slightly higher with the 150 mg dose of esmethadone, but no significant differences from placebo were observed.

Considering that MDD is a highly prevalent and life-threatening condition and available treatments have limited and delayed efficacy and metabolic and neurological side effects, an unmet need exists for safe, well-tolerated, and rapidly effective antidepressants. Patients in need should not have restricted or delayed access to potentially safe, well-tolerated, and life-saving drugs [36].

In conclusion, the results of these two HAP studies, in accordance with results in preclinical models [30, 59], indicate no meaningful abuse potential for esmethadone at all tested doses. These results confirm a recent Drug Enforcement Administration (DEA) publication stating: “The d-isomer lacks significant respiratory depressant action and addiction liability…” [65]. If Phase 3 results confirm the rapid, robust, and sustained antidepressant effects and the favorable tolerability and safety profiles of the Phase 2 study, esmethadone has the potential to become an important first-line adjunctive treatment for MDD.

## Supplementary information

Supplemental Material
