# Supplementary material for: The novel uncompetitive NMDA receptor antagonist esmethadone (REL-1017) has no meaningful abuse potential in recreational drug users
Source: Transl Psychiatry. 2023 Jun 7;13:192. doi: 10.1038/s41398-023-02473-8 (PMC10247777; doi:10.1038/s41398-023-02473-8)
Supplement: Supplementary file 1 — Supplemental Material [file 41398_2023_2473_MOESM1_ESM.docx]

**Supplemental Material**

Supplemental Figure 1. Overview of study design for Oxycodone Study


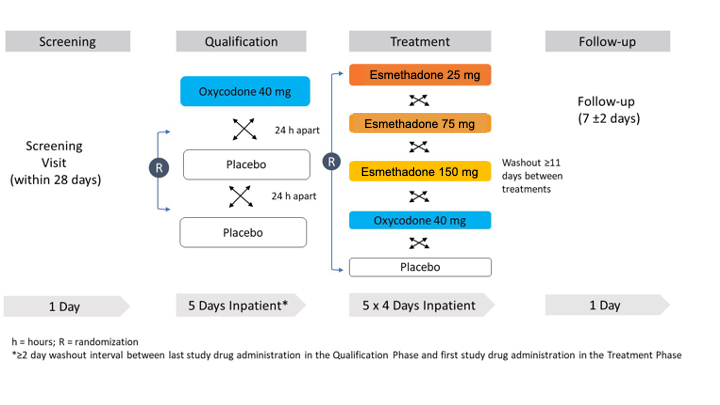


Supplemental Figure 2. Overview of study design for Ketamine Study


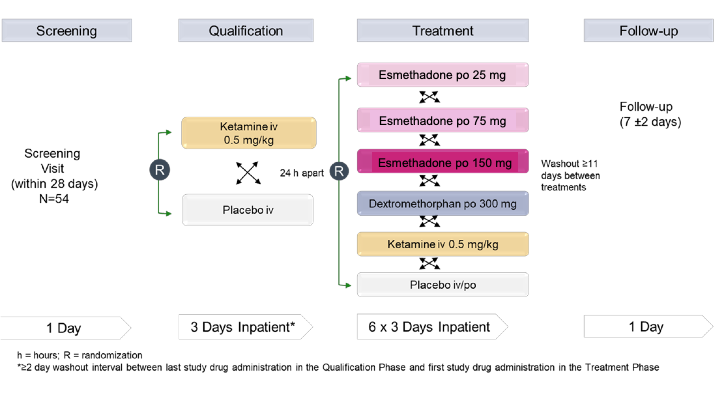


Supplemental Figure 3. Mean (SE) Drug Liking VAS scores over 24 hours post dose (Completer Population) in the Oxycodone Study (top) and the Ketamine Study (bottom)


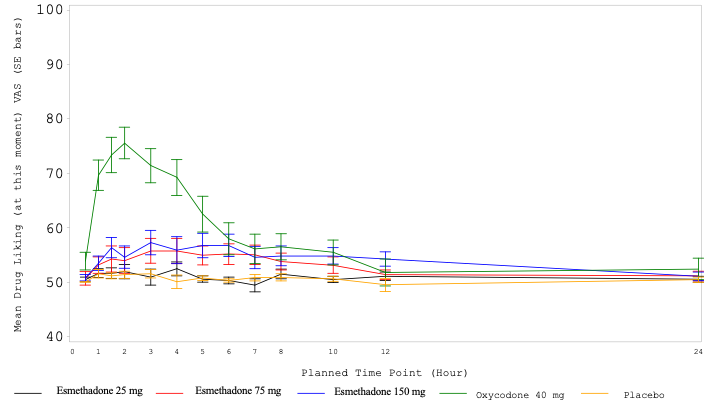


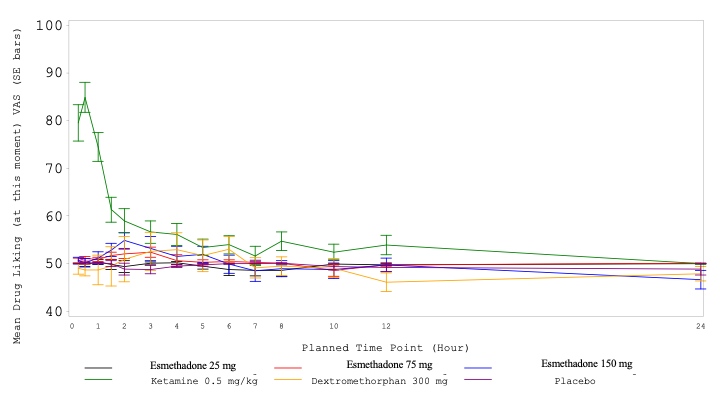


Supplemental Figure 4. Mean (SE) Plasma Concentrations for Esmethadone and Oxycodone Over Time (PK population)


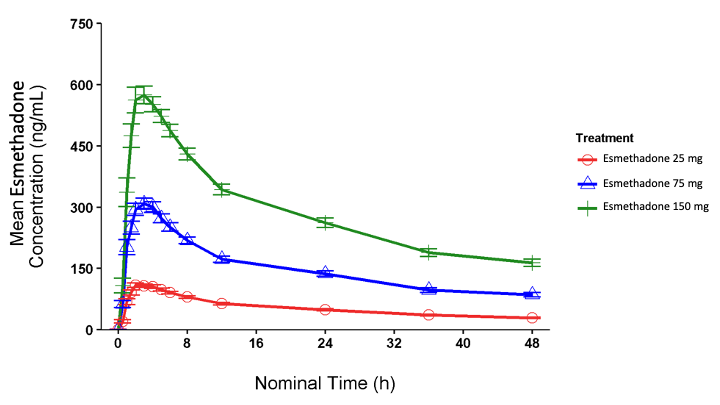


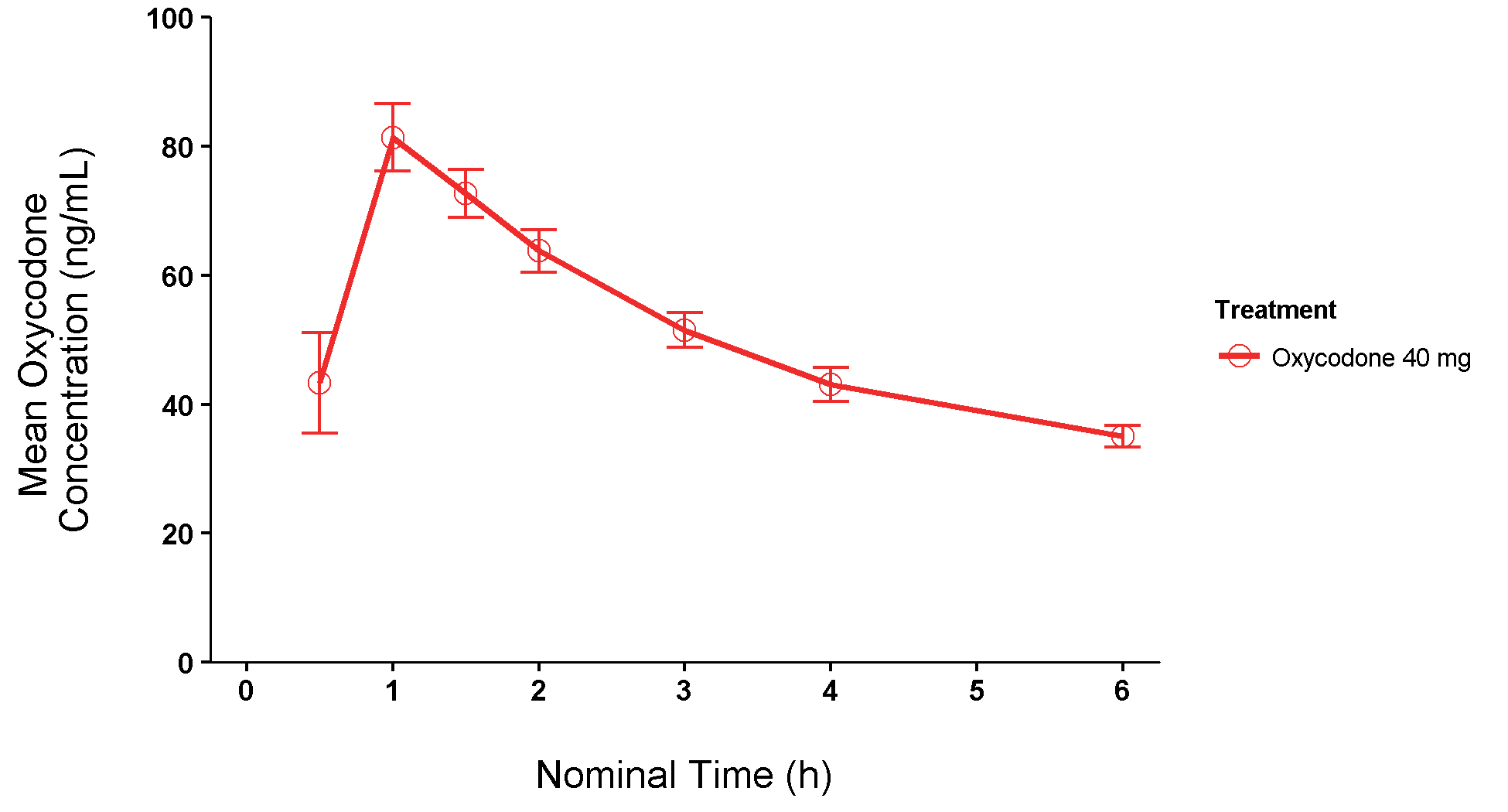


Supplemental Figure 5. Mean (SE) Plasma Concentrations for Esmethadone and Ketamine Over Time (PK population)


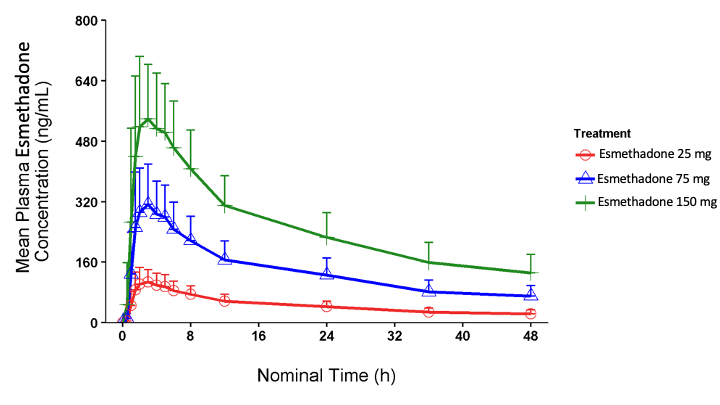


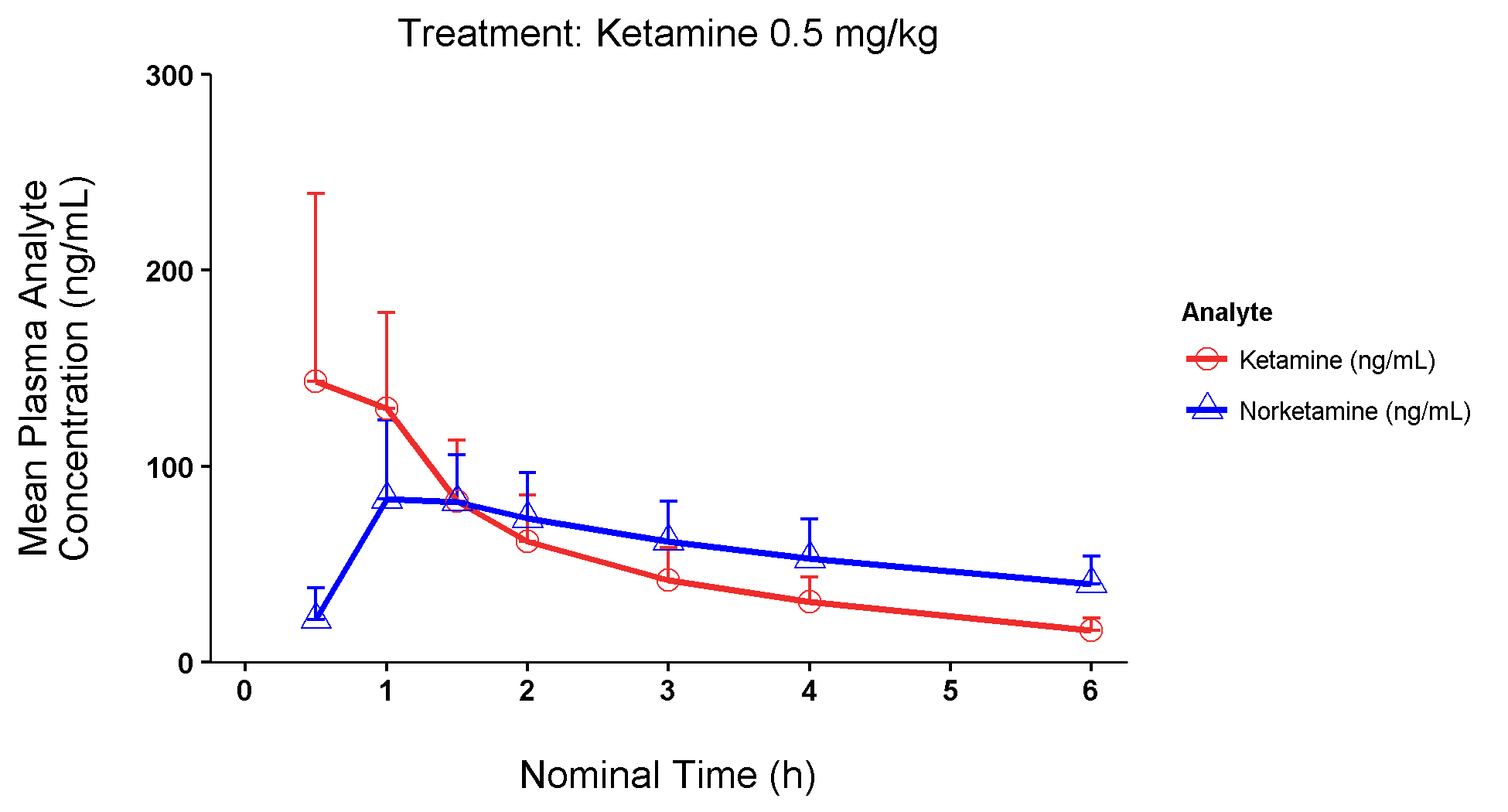


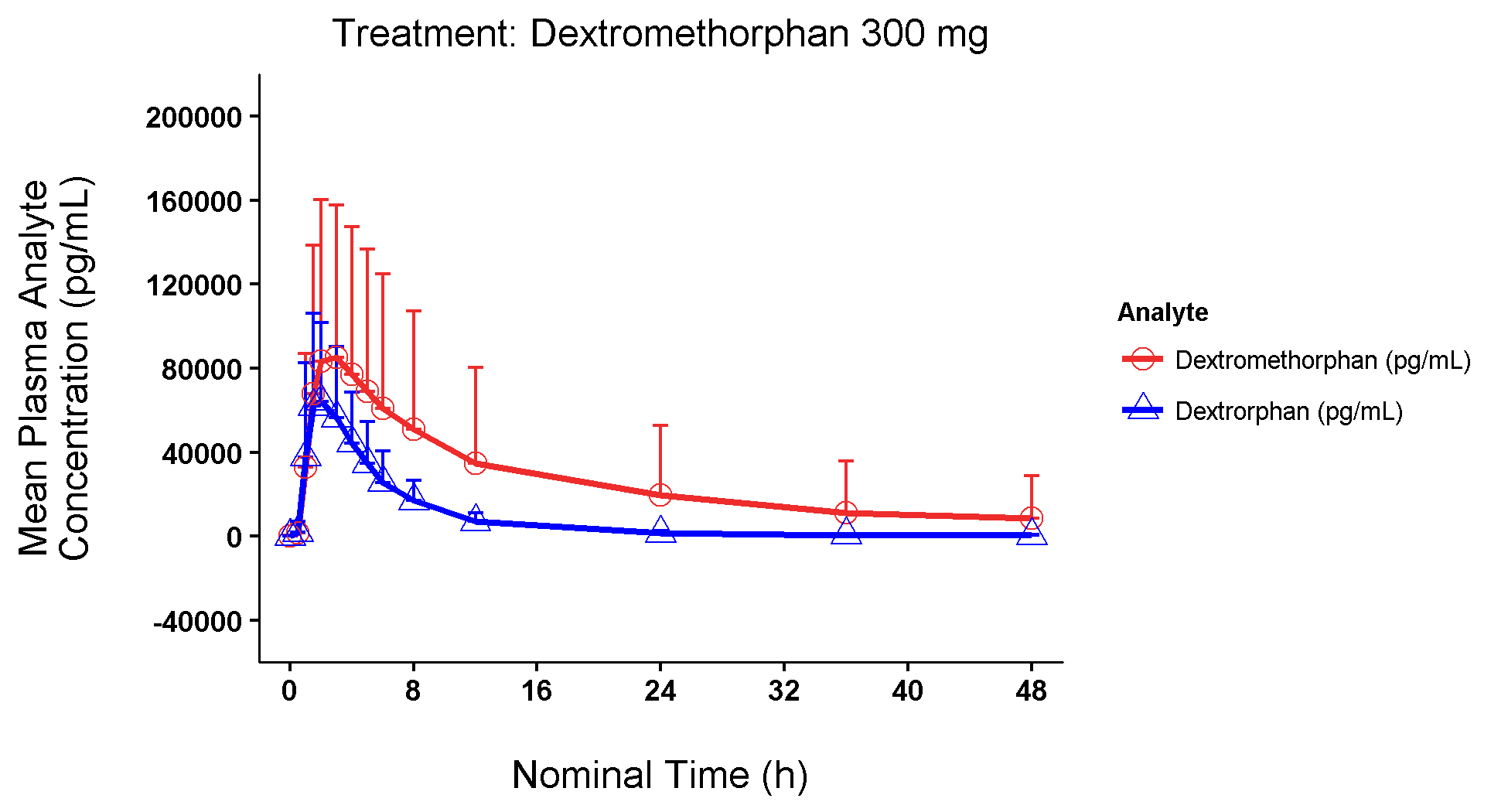


Supplemental Table 1. Descriptive Statistics of Drug Liking VAS E_max_ – Oxycodone Study (Modified Completer Population)

| Statistic | Esmethadone  25 mg (N=44) | Esmethadone  75 mg (N=44) | Esmethadone  150 mg (N=44) | Oxycodone  40 mg (N=44) | Placebo (N=44) |
| --- | --- | --- | --- | --- | --- |
| Mean (SD) | 53.0 (8.67) | 58.2 (14.98) | 64.9 (16.74) | 85.0 (15.40) | 51.7 (4.28) |
| Median | 50.0 | 50.0 | 58.0 | 89.0 | 50.0 |
| Range | 50 – 100 | 50 – 100 | 50 – 100 | 50 – 100 | 50 – 70 |

E_max_=maximum effect; range=minimum – maximum; SD=standard deviation; VAS=visual analog scale.

Drug Liking VAS is a bipolar scale where a score of 0 represents “strong disliking,” a score of 100 represents “strong liking,” and a score of 50 represents “neither like nor dislike” (neutral point). The question text is, “At this moment, my liking for this drug is.”

Supplemental Table 2. Inferential Analysis Results for Drug Liking VAS E_max_ – Oxycodone Study (Modified Completer Population)

| Pairwise Comparisons | Modified Completer Population | | |
| --- | --- | --- | --- |
|  | Mean/ Median of  Intra‑participant Difference | 95% CI/ Quartiles | **P‑value** |
| **Study Validity** |  |  |  |
| Oxycodone 40 mg – Placebo | 36.0 | (20.0, 49.5)^b^ | **<0.001** |
| **Relative Abuse Potential** |  |  |  |
| Oxycodone 40 mg – Esmethadone 25 mg | 34.5 | (19.5, 48.5)^b^ | **<0.001** |
| Oxycodone 40 mg – Esmethadone 75 mg | 26.8 | (22.0, ∞)^a^ | **<0.001** |
| Oxycodone 40 mg – Esmethadone 150 mg | 19.0 | (9.0, 34.5) ^b^ | **<0.001** |
| **Absolute Abuse Potential** |  |  |  |
| Esmethadone 25 mg – Placebo | 0.0 | (0.0, 1.0)^b^ | **<0.001** |
| Esmethadone 75 mg – Placebo | 0.0 | (‑0.5, 5.0)^b^ | **<0.001** |
| Esmethadone 150 mg – Placebo | 8.0 | (0.0, 24.5)^b^ | 0.082 |

CI=confidence interval; E_max_=maximum effect, VAS=visual analog scale.

Note: Friedman's test was used to assess overall treatment effects: p value <0.001 for both populations.

Study validity hypothesis: H_o_: µ_C_ – µ_P_ ≤ 15 vs. H_a_: µ_C_ – µ_P_ > 15; 1‑sided test (α = 0.05)

Relative abuse potential hypothesis: H_o_: µ_C_ – µ_T_ ≤ 0 vs. H_a_: µ_C_ – µ_T_ > 0; 1‑sided test (α = 0.05)

Absolute abuse potential hypothesis: H_o_: µ_T_ – µ_P_ ≥ 11 vs. H_a_: µ_T_ – µ_P_ < 11; 1‑sided test (α = 0.05)

where P = placebo; C = positive control; and T = test drug.

a Paired *t* test was used to assess the mean difference between the 2 treatments; mean and 95% CI are presented.

b Sign test was used to assess the median difference between the 2 treatments; median and quartiles are presented.

Bolded p‑values are statistically significant. A statistically significant p‑value for the comparison of esmethadone vs. placebo indicates that esmethadone at that dose level has a similar response profile to placebo.

Supplemental Table 3. Descriptive Statistics of Drug Liking VAS E_max_ – Ketamine Study (Modified Completer Population)

| Statistic | Esmethadone  25 mg (N=50) | Esmethadone  75 mg (N=50) | Esmethadone  150 mg (N=50) | Ketamine  0.5 mg/kg (N=50) | DXM  300 mg (N=50) | Placebo (N=50) |
| --- | --- | --- | --- | --- | --- | --- |
| Mean (SD) | 51.4 (3.31) | 55.0 (9.66) | 59.4 (14.46) | 90.8 (13.48) | 68.8 (18.38) | 51.0 (2.25) |
| Median | 50.0 | 50.5 | 51.0 | 100.0 | 61.0 | 50.0 |
| Range | 50 – 66 | 50 – 100 | 50 – 100 | 50 – 100 | 50 – 100 | 50 – 63 |

DXM=dextromethorphan; E_max_=maximum effect; range=minimum – maximum; SD=standard deviation; VAS=visual analog scale.

Drug Liking VAS is a bipolar scale where a score of 0 represents “strong disliking,” a score of 100 represents “strong liking,” and a score of 50 represents “neither like nor dislike” (neutral point). The question text is, “At this moment, my liking for this drug is.”

Supplemental Table 4. Inferential Analysis Results for Drug Liking VAS E_max_ – Ketamine Study (Modified Completer Population)

| Pairwise Comparisons | Modified Completer Population | | |
| --- | --- | --- | --- |
|  | Mean/Median of  Intra‑participant Difference | 95% CI/ Quartiles | **P-value** |
| **Study Validity** |  |  |  |
| Ketamine 0.5 mg/kg – Placebo | 49.0 | (27.0, 50.0)b | **<0.001** |
| **Relative Abuse Potential** |  |  |  |
| Ketamine 0.5 mg/kg – Esmethadone 25 mg | 48.5 | (29.0, 50.0)b | **<0.001** |
| Ketamine 0.5 mg/kg – Esmethadone 75 mg | 42.5 | (25.0, 50.0)b | **<0.001** |
| Ketamine 0.5 mg/kg – Esmethadone 150 mg | 34.5 | (22.0, 49.0)b | **<0.001** |
| **Absolute Abuse Potential** |  |  |  |
| Esmethadone 25 mg – Placebo | 0.0 | (0.0, 0.0)b | **<0.001** |
| Esmethadone 75 mg – Placebo | 0.0 | (0.0, 3.0)b | **<0.001** |
| Esmethadone 150 mg – Placebo | 0.0 | (0.0, 14.0)b | **0.005** |
| **Exploratory Comparisons** |  |  |  |
| DXM 300 mg – Placebo | 8.0 | (0.0, 35.0)b | 0.444 |
| DXM 300 mg – Esmethadone 25 mg | 10.0 | (0.0, 34.0)b | **<0.001** |
| DXM 300 mg – Esmethadone 75 mg | 13.8 | (8.7, ∞)a | **<0.001** |
| DXM 300 mg – Esmethadone 150 mg | 9.3 | (4.2, ∞)a | **0.002** |
| Ketamine 0.5 mg/kg – DXM 300 mg | 22.0 | (17.5, ∞)a | **<0.001** |

CI=confidence interval; DXM=dextromethorphan; E_max_=maximum effect; VAS=visual analog scale.

Note: Friedman's test was used to assess overall treatment effects: p value <0.001 for both populations.

Study validity: H_o_: µ_C1_ – µ_P_ ≤ 15 vs. H_a_: µ_C1_ – µ_P_ > 15; 1-sided test (α = 0.05).

Relative abuse potential: H_o_: µ_C1_ – µ_T_ ≤ 0 vs. H_a_: µ_C1_ – µ_T_ > 0; 1-sided test (α = 0.05).

Absolute abuse potential: H_o_: µ_T_ – µ_P_ ≥ 11 vs. H_a_: µ_T_ – µ_P_ < 11; 1-sided test (α = 0.05).

a Paired *t* test was used to assess the mean difference between the 2 treatments; mean and 95% CI are presented.

b Sign test was used to assess the median difference between the 2 treatments; median and quartiles are presented.

Bolded p‑values are statistically significant. A statistically significant p-value for the comparison of esmethadone vs. placebo indicates that esmethadone at that dose level has a similar response profile to placebo.

Supplemental Table 5. Descriptive Statistics for Secondary Endpoints– Oxycodone Study (Modified Completer Population)

| Statistic | Esmethadone  25 mg (N=44) | Esmethadone  75 mg (N=44) | Esmethadone  150 mg (N=44) | Oxycodone  40 mg (N=44) | Placebo (N=44) |
| --- | --- | --- | --- | --- | --- |
| *Overall Drug Liking VAS* | | | | | |
| Mean (SD) | 51.8 (7.02) | 58.5 (19.54) | 61.5 (18.83) | 75.1 (23.10) | 51.3 (10.98) |
| Median | 50.0 | 50.0 | 50.5 | 73.5 | 50.0 |
| Range | 36 – 80 | 12 – 100 | 16 – 100 | 9 – 100 | 0 – 98 |
| *Take Drug Again VAS* | | | | | |
| Mean (SD) | 51.1 (16.31) | 57.7 (23.84) | 61.3 (23.35) | 77.1 (25.85) | 49.7 (15.67) |
| Median | 50.0 | 50.0 | 50.0 | 86.0 | 50.0 |
| Range | 0 – 100 | 0 – 100 | 0 – 100 | 7 – 100 | 0 – 100 |
| *High VAS* | | | | | |
| E_max_ |  |  |  |  |  |
| Mean (SD) | 7.5 (19.83) | 20.3 (30.53) | 31.3 (35.11) | 75.8 (26.08) | 5.0 (13.36) |
| Median | 0.0 | 1.0 | 16.0 | 83.0 | 0.0 |
| Range | 0 – 100 | 0 – 100 | 0 – 100 | 5 – 100 | 0 – 59 |
| *Good Effects VAS* | | | | | |
| E_max_ |  |  |  |  |  |
| Mean (SD) | 7.5 (20.88) | 21.2 (31.52) | 33.1 (36.34) | 75.7 (24.96) | 7.0 (18.73) |
| Median | 0.0 | 0.0 | 19.0 | 81.0 | 0.0 |
| Range | 0 – 100 | 0 – 100 | 0 – 100 | 7 – 100 | 0 – 96 |
| *Bad Effects VAS* | | | | | |
| E_max_ |  |  |  |  |  |
| Mean (SD) | 1.7 (6.00) | 7.9 (21.96) | 13.6 (25.38) | 25.8 (28.82) | 0.8 (4.22) |
| Median | 0.0 | 0.0 | 0.0 | 14.0 | 0.0 |
| Range | 0 – 28 | 0 – 100 | 0 – 89 | 0 – 87 | 0 – 28 |
| *Alertness/Drowsiness VAS* | | | | | |
| E_min_ |  |  |  |  |  |
| Mean (SD) | 45.2 (10.07) | 40.6 (15.08) | 34.0 (16.61) | 18.0 (15.36) | 46.7 (11.78) |
| Median | 50.0 | 50.0 | 36.5 | 16.0 | 50.0 |
| Range | 0 – 50 | 0 – 50 | 0 – 54 | 0 – 50 | 0 – 90 |
| *Any Effects VAS* | | | | | |
| E_max_ |  |  |  |  |  |
| Mean (SD) | 8.2 (21.17) | 24.9 (33.46) | 37.3 (37.12) | 79.3 (25.69) | 5.4 (14.20) |
| Median | 0.0 | 3.5 | 26.0 | 85.0 | 0.0 |
| Range | 0 – 100 | 0 – 100 | 0 – 100 | 5 – 100 | 0 – 69 |
| *Drug Similarity VAS - Opioids* | | | | | |
| n | 43 | 41 | 44 | 44 | 44 |
| Mean (SD) | 4.1 (14.63) | 15.9 (29.81) | 35.2 (39.07) | 81.1 (31.57) | 6.4 (15.94) |
| Median | 0.0 | 0.0 | 15.0 | 100.0 | 0.0 |
| Range | 0 – 90 | 0 – 100 | 0 – 100 | 0 – 100 | 0 – 76 |

E_max_=maximum effect; range=minimum – maximum; SD=standard deviation; VAS=visual analog scale.

Supplemental Table 6. Descriptive Statistics for Secondary Endpoints – Ketamine Study (Modified Completer Population)

| Statistic | Esmethadone  25 mg (N=50) | Esmethadone  75 mg (N=50) | Esmethadone 150 mg (N=50) | Ketamine 0.5 mg/kg (N=50) | DXM  300 mg (N=50) | Placebo (N=50) | | |
| --- | --- | --- | --- | --- | --- | --- | --- | --- |
| *Overall Drug Liking VAS* | | | | | | | | |
| Mean (SD) | 51.3 (8.06) | 50.8 (13.86) | 53.1 (20.28) | 87.6 (19.52) | 58.9 (30.00) | 47.7 (9.77) | | |
| Median | 50.0 | 50.0 | 50.0 | 100.0 | 59.0 | 50.0 | | |
| Range | 43 – 100 | 0 – 100 | 0 – 100 | 41 – 100 | 0 – 100 | 0 – 54 | | |
| *Take Drug Again VAS* | | | | | | | | |
| Mean (SD) | 50.5 (10.98) | 50.0 (18.44) | 53.5 (24.64) | 88.5 (22.08) | 56.3 (31.78) | 47.8 (11.22) | | |
| Median | 50.0 | 50.0 | 50.0 | 100.0 | 53.5 | 50.0 | | |
| Range | 0 – 100 | 0 – 100 | 0 – 100 | 1 – 100 | 0 – 100 | 0 – 75 | | |
| *High VAS* | | | | | | | |  |
| E_max_ |  |  |  |  |  | | |  |
| Mean (SD) | 2.7 (6.40) | 10.4 (19.10) | 16.9 (26.05) | 87.5 (21.85) | 60.0 (36.62) | | | 2.1 (4.30) |
| Median | 0.0 | 0.0 | 4.0 | 100.0 | 73.0 | | | 0.0 |
| Range | 0 – 27 | 0 – 79 | 0 – 100 | 19 – 100 | 1 – 100 | | | 0 – 19 |
| *Good Effects VAS* | | | | | | | |  |
| E_max_ |  |  |  |  |  | | |  |
| Mean (SD) | 3.0 (8.81) | 10.4 (20.52) | 19.5 (28.88) | 87.4 (21.02) | 48.0 (35.43) | | | 2.8 (8.71) |
| Median | 0.0 | 0.0 | 5.0 | 100.0 | 50.0 | | | 0.0 |
| Range | 0 – 50 | 0 – 100 | 0 -100 | 14 – 100 | 0 – 100 | | | 0 – 57 |
| *Bad Effects VAS* | | | | | | | |  |
| Emax |  |  |  |  |  | | |  |
| Mean (SD) | 2.7 (11.74) | 5.3 (14.67) | 6.8 (18.22) | 13.3 (28.22) | 33.4 (36.66) | | | 3.3 (9.27) |
| Median | 0.0 | 0.0 | 0.0 | 0.0 | 18.5 | | | 0.0 |
| Range | 0 – 80 | 0 – 71 | 0 – 90 | 0 – 100 | 0 – 100 | | | 0 – 50 |
| *Alertness/Drowsiness VAS* | | | | | | |  | |
| E_max_ |  |  |  |  |  | |  | |
| Mean (SD) | 51.4 (7.09) | 52.7 (9.60) | 53.7 (10.08) | 69.4 (21.33) | 59.0 (15.94) | | 51.2 (7.05) | |
| Median | 50.0 | 50.0 | 50.0 | 56.5 | 50.0 | | 50.0 | |
| Range | 50 – 100 | 50 – 100 | 50 – 100 | 50 – 100 | 50 – 100 | | 50 – 100 | |
| *Any Effects VAS* | | | | | | |  | |
| E_max_ |  |  |  |  |  | |  | |
| Mean (SD) | 4.2 (11.65) | 13.7 (21.65) | 21.2 (28.72) | 89.9 (20.27) | 66.2 (35.76) | | 4.4 (8.10) | |
| Median | 0.0 | 1.5 | 7.5 | 100.0 | 77.5 | | 1.0 | |
| Range | 0 – 73 | 0 – 89 | 0 – 100 | 11 – 100 | 1 – 100 | | 0 – 36 | |
| *Hallucinations VAS* | | | | | | |  | |
| E_max_ |  |  |  |  |  | |  | |
| Mean (SD) | 0.2 (0.56) | 0.3 (0.78) | 0.6 (2.26) | 23.6 (38.29) | 7.6 (19.38) | | 0.2 (0.44) | |
| Median | 0.0 | 0.0 | 0.0 | 0.0 | 0.0 | | 0.0 | |
| Range | 0 – 3 | 0 – 5 | 0 – 14 | 0 – 100 | 0 – 100 | | 0 – 2 | |
| *Bowdle VAS – External Perception* | | | | | | |  | |
| E_max_ |  |  |  |  |  | |  | |
| Mean (SD) | 0.2 (0.58) | 1.1 (3.24) | 1.9 (4.61) | 34.1 (31.88) | 10.9 (17.93) | | 0.2 (0.63) | |
| Median | 0.0 | 0.0 | 0.1 | 21.3 | 2.3 | | 0.0 | |
| Range | 0 – 4 | 0 – 18 | 0 – 21 | 0 – 100 | 0 – 100 | | 0 – 4 | |
| *Bowdle VAS – Internal Perception* | | | | | | | | |
| E_max_ |  |  |  |  |  | |  | |
| Mean (SD) | 0.1 (0.17) | 0.4 (1.05) | 0.2 (0.59) | 17.4 (24.51) | 6.6 (10.52) | | 0.3 (1.37) | |
| Median | 0.0 | 0.0 | 0.0 | 8.7 | 0.9 | | 0.0 | |
| Range | 0 – 1 | 0 – 5 | 0 – 3 | 0 – 100 | 0 – 40 | | 0 – 10 | |
| *Drug Similarity VAS* | | | | | | | | |
| Ketamine |  |  |  |  |  | |  | |
| n | 50 | 50 | 50 | 49 | 50 | | 50 | |
| Mean (SD) | 0.5 (2.36) | 5.7 (15.39) | 10.2 (24.42) | 93.0 (21.85) | 38.6 (36.37) | | 1.2 (6.86) | |
| Median | 0.0 | 0.0 | 0.0 | 100.0 | 34.5 | | 0.0 | |
| Range | 0 – 14 | 0 – 85 | 0 – 100 | 0 – 100 | 0 – 100 | | 0 – 48 | |
| Opioids |  |  |  |  |  | |  | |
| n | 29 | 29 | 29 | 28 | 29 | | 29 | |
| Mean (SD) | 6.0 (19.36) | 12.1 (28.96) | 17.2 (29.42) | 22.2 (34.84) | 24.6 (34.01) | | 5.6 (19.92) | |
| Median | 0.0 | 0.0 | 0.0 | 3.0 | 0.0 | | 0.0 | |
| Range | 0 – 100 | 0 – 98 | 0 – 100 | 0 – 100 | 0 – 100 | | 0 – 100 | |

DXM=dextromethorphan; E_max_=maximum effect; range=minimum – maximum; SD=standard deviation; VAS=visual analog scale.

Supplemental Table 7. Noncompartmental PK parameters for Esmethadone from Oxycodone Study and Ketamine Study (PK Population)

|  | Oxycodone Study | | | Ketamine Study | | |
| --- | --- | --- | --- | --- | --- | --- |
|  | Esmethadone 25 mg  (N=46) | Esmethadone 75 mg  (N=49) | Esmethadone 150 mg  (N=49) | Esmethadone 25 mg  (N=52) | Esmethadone 75 mg  (N=52) | Esmethadone 150 mg  (N=52) |
| **C_max_ (ng/mL)** |  |  |  |  |  |  |
| Geometric Mean | 120 | 326 | 631 | 110 | 325 | 590 |
| Geometric CV% | 26.5 | 34.3 | 26.7 | 36.7 | 35.6 | 26.5 |
| Range | 64.8 – 220 | 91.2 – 567 | 384 – 1330 | 44.7 – 205 | 143 – 879 | 320 – 1080 |
| **T_max_ (hour)** |  |  |  |  |  |  |
| Median | 2.1 | 3.1 | 3.1 | 2.9 | 2.9 | 1.9 |
| Range | 1.1 – 6.2 | 1.0 – 8.6 | 1.1 – 5.1 | 1.4 – 7.9 | 1.4 – 5.9 | 0.9 – 7.9 |
| **AUC_0‑last_ (h*ng/mL)** | |  |  |  |  |  |
| Geometric Mean | 2420 | 6690 | 13 200 | 2120 | 6220 | 11 600 |
| Geometric CV% | 31.7 | 38.2 | 28.6 | 33.8 | 34.0 | 29.0 |
| Range | 1230 – 4250 | 1570 – 12 100 | 6830 – 22 700 | 863 – 4010 | 2610 – 13 100 | 5900 – 20 300 |

AUC_0‑last_=area under the curve from time zero to last assessment; C_max_=maximum observed concentration; CV%=percent coefficient of variation; PK=pharmacokinetic; range=minimum – maximum; T_max_=time of occurrence of C_max_.

Supplemental Table 8. Incidence of TEAEs occurring in at least 5% or subjects in any group (Safety Population)

|  | Oxycodone Study | | | | | Ketamine Study | | | | | |
| --- | --- | --- | --- | --- | --- | --- | --- | --- | --- | --- | --- |
|  | Esmethadone 25 mg (N=47) | Esmethadone 75 mg (N=49) | Esmethadone 150 mg (N=49) | Oxycodone 40 mg (N=48) | Placebo (N=49) | Esmethadone 25 mg  (N=53) | Esmethadone 75 mg  (N=53) | Esmethadone 150 mg  (N=53) | Ketamine 0.5 mg/kg  (N=52) | DXM 300 mg  (N=54) | Placebo  (N=52) |
| Number of TEAEs | 10 | 21 | 28 | 80 | 8 | 23 | 32 | 30 | 16 | 105 | 24 |
| Any TEAE | 6 (12.8) | 6 (12.2) | 14 (28.6) | 25 (52.1) | 6 (12.2) | 13 (24.5) | 22 (41.5) | 21 (39.6) | 13 (25.0) | 40 (74.1) | 16 (30.8) |
| Treatment‑related TEAEs | 7 | 15 | 21 | 75 | 4 | 6 | 15 | 20 | 9 | 87 | 11 |
| Any treatment‑related AE | 5 (10.6) | 6 (12.2) | 11 (22.4) | 25 (52.1) | 4 (8.2) | 5 (9.4) | 11 (20.8) | 13 (24.5) | 8 (15.4) | 36 (66.7) | 7 (13.5) |
| Nausea | 1 (2.1) | 4 (8.2) | 6 (12.2) | 14 (29.2) | 0 | 0 | 5 (9.4) | 10 (18.9) | 1 (1.9) | 25 (46.3) | 0 |
| Vomiting | 1 (2.1) | 2 (4.1) | 3 (6.1) | 9 (18.8) | 0 | 0 | 1 (1.9) | 2 (3.8) | 0 | 16 (29.6) | 0 |
| Dizziness | 0 | 0 | 0 | 5 (10.4) | 0 | 0 | 1 (1.9) | 1 (1.9) | 1 (1.9) | 6 (11.1) | 1 (1.9) |
| Headache | 4 (8.5) | 2 (4.1) | 4 (8.2) | 1 (2.1) | 2 (4.1) | 8 (15.1) | 9 (17.0) | 4 (7.5) | 9 (17.3) | 10 (18.5) | 10 (19.2) |
| Somnolence | 0 | 2 (4.1) | 3 (6.1) | 10 (20.8) | 1 (2.0) | 2 (3.8) | 3 (5.7) | 2 (3.8) | 2 (3.8) | 4 (7.4) | 2 (3.8) |
| Pruritus | 0 | 1 (2.0) | 1 (2.0) | 10 (20.8) | 1 (2.0) | 1 (1.9) | 1 (1.9) | 2 (3.8) | 0 | 4 (7.4) | 0 |
| Hot flush | 0 | 0 | 0 | 3 (6.3) | 0 | 0 | 0 | 0 | 0 | 0 | 0 |

DXM=dextromethorphan; TEAE=treatment-emergent adverse event

For each row category and at a given treatment at onset, a participant with 2 or more adverse events in

that category/treatment is counted only once.

Treatment‑related TEAEs were those considered possibly, probably and definitely related to study treatment.

The number and percentage of participants with TEAEs / treatment‑related TEAEs / serious TEAEs were summarized by system organ class and preferred term for each treatment at onset. TEAEs were also summarized by maximum severity and maximum relationship for each treatment at onset.

Supplemental Table 9. Mean (standard deviation) QTcF Interval at baseline, 2 hours post dose, and change from baseline – Oxycodone Study (Safety Population)

|  | Mean (Standard Deviation) QTcF Interval (ms) | | | | |
| --- | --- | --- | --- | --- | --- |
|  | Esmethadone | | | Oxycodone |  |
|  | 25 mg (N=47) | 75 mg (N=49) | 150 mg  (N=49) | 40 mg  (N=48 | Placebo  (N=49 |
| Baseline | 400.5 (13.8) | 399.4 (15.4) | 401.7 (15.8) | 400.1 (14.8) | 403.0 (15.3) |
| 2 hours | 404.4 (14.5) | 406.2 (14.8) | 410.8 (16.9) | 411.6 (17.6) | 401.7 (14.5) |
| Change from baseline to 2 hours | 3.9 (8.2) | 6.9 (8.2) | 9.1 (11.0) | 11.4 (13.7) | -1.3 (11.0) |
